# Electric coupling between distant nitrate reduction and sulfide oxidation in marine sediment

**DOI:** 10.1038/ismej.2014.19

**Published:** 2014-02-27

**Authors:** Ugo Marzocchi, Daniela Trojan, Steffen Larsen, Rikke Louise Meyer, Niels Peter Revsbech, Andreas Schramm, Lars Peter Nielsen, Nils Risgaard-Petersen

**Affiliations:** 1 Section for Microbiology, Department of Bioscience, Aarhus University, Aarhus, Denmark; 2 Center for Geomicrobiology, Department of Bioscience, Aarhus University, Aarhus, Denmark; 3 Interdisciplinary Nanoscience Center, Aarhus University, Aarhus, Denmark; 4 Aarhus Institute of Advanced Studies, Aarhus University, Aarhus, Denmark

**Keywords:** nitrate, cable bacteria, electric current, cathodic reduction, sediment

## Abstract

Filamentous bacteria of the *Desulfobulbaceae* family can conduct electrons over centimeter-long distances thereby coupling oxygen reduction at the surface of marine sediment to sulfide oxidation in deeper anoxic layers. The ability of these cable bacteria to use alternative electron acceptors is currently unknown. Here we show that these organisms can use also nitrate or nitrite as an electron acceptor thereby coupling the reduction of nitrate to distant oxidation of sulfide. Sulfidic marine sediment was incubated with overlying nitrate-amended anoxic seawater. Within 2 months, electric coupling of spatially segregated nitrate reduction and sulfide oxidation was evident from: (1) the formation of a 4–6-mm-deep zone separating sulfide oxidation from the associated nitrate reduction, and (2) the presence of pH signatures consistent with proton consumption by cathodic nitrate reduction, and proton production by anodic sulfide oxidation. Filamentous *Desulfobulbaceae* with the longitudinal structures characteristic of cable bacteria were detected in anoxic, nitrate-amended incubations but not in anoxic, nitrate-free controls. Nitrate reduction by cable bacteria using long-distance electron transport to get privileged access to distant electron donors is a hitherto unknown mechanism in nitrogen and sulfur transformations, and the quantitative importance for elements cycling remains to be addressed.

## Introduction

Electric currents can couple cathodic O_2_ reduction (O_2_+4H^+^+4e^−^→2H_2_O) at the surface of marine sediment to anodic oxidation of sulfide (H_2_S+4H_2_O→SO_4_^2−^+10H^+^+8e^−^) over distances of more than 1 cm (Nielsen *et al.*, 2010; Risgaard-Petersen *et al.*, 2012). Evidence for electric coupling between these spatially segregated half-cell reactions includes (1) the formation of oxygen- and sulfide-depleted zones in the absence of reactive Mn and Fe oxides and mixing, and (2) the appearance of a distinct pH maximum in the oxic zone and a minimum in the anoxic zone, in accordance with proton consumption by cathodic O_2_ reduction and proton production by anodic sulfide oxidation, respectively. Recently, Pfeffer *et al.* (2012) showed that the electric coupling between spatially segregated half-cell reactions was mediated by filamentous, multicellular bacteria belonging to the family *Desulfobulbaceae*. These ‘cable bacteria' have uniform ridges formed by strings located inside a periplasmic space that is continuous between the individual cells. The strings have distinct electronic properties comparable to electron conductors, and they are proposed to be electric wires with the surrounding cytoplasmic and periplasmic membranes serving as insulation (Pfeffer *et al.*, 2012).

The presence of cable bacteria that act as electron conductors and allow redox couples to interact far beyond their physical presence promotes a sediment geochemistry that cannot be understood with classical geochemical models (Risgaard-Petersen *et al.*, 2012). However, the occurrence of these organisms and their impact on sediment geochemistry has been addressed only in the presence of O_2_, and to date, it remains unknown whether other electron acceptors such as NO_3_^−^ can be used. Thermodynamically, NO_3_^−^ is an electron acceptor almost as good as O_2_ and several prokaryotes are able to switch from O_2_ to NO_3_^−^ respiration when anoxic conditions occur. Also, the closest cultured relative of cable bacteria, *Desulfobulbus propionicus*, can grow with NO_3_^−^ as an electron acceptor and can couple sulfide oxidation to NO_2_^−^ reduction (Dannenberg *et al.*, 1992).

In this study we investigated whether cathodic NO_3_^−^ reduction can sustain the distant oxidation of sulfide in marine sediment as previously described for O_2_ (Nielsen *et al.*, 2010). In a first set of experiments, we incubated sediment from Aarhus Bay under anoxic seawater containing 200–230 μM NO_3_^−^ and assessed the development of the traits typical of the electric coupling between spatially segregated half-cell reactions. Successively, we ran parallel incubations to compare NO_3_^−^ and O_2_ effectiveness in sustaining the distant oxidation of sulfide. Finally, we addressed the nature of the conductors investigating whether cable bacteria can grow under anoxic conditions in the presence of NO_3_^−^.

## Materials and methods

### Sediment sampling and pre-treatment

Intact sediment samples were collected from Aarhus Bay (Denmark) at station M5 (56°06′20′′N, 10°27′48′′E; depth 30 m) using a box corer. On board, the upper 10–12 cm of sediment were discarded to minimize the presence of metal oxides and exclude large burrowing animals. The underlying sulfidic sediment was sealed in airtight bags, brought to the laboratory and stored at 15 °C. Within a few weeks, the bags were opened and the sediment was sieved (sieve mesh size 0.5 mm), homogenized and packed into glass liners or chambers before being incubated. Sediment exposure to air was minimized throughout the handling procedures.

### Sediment incubation with anoxic, NO_3_^−^-amended overlying water

**Figure 1:**
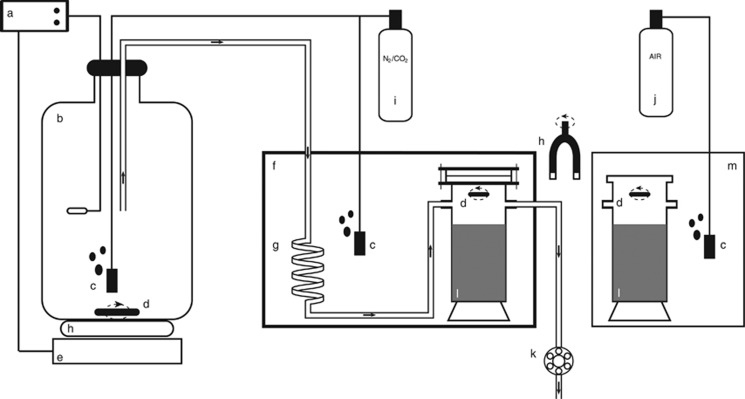
The flow-through system. Arrows indicate the direction of water flow. (**a**) Thermostat; (**b**) 20-liter water reservoir maintained at 30 °C; (**c**) gas-diffusing stones; (**d**) stirrer (teflon-coated magnetic bars); (**e**) heater; (**f**) sealed anoxic aquarium maintained at 13.6 °C; (**g**) coil for cooling of inflow water; (**h**) rotating magnet driving the stirrers; (**i**) N_2_ (99.96%) and CO_2_ (0.04%) gas mix tank; (**j**) air tank; (**k**) peristaltic pump; (**l**) sediment; (**m**) aerated aquarium.

To address whether NO_3_^−^ reduction can sustain distant oxidation of sulfide, the sediment was incubated in a modified version of the flow-through system described by Risgaard-Pedersen *et al.* (1994), where both the gas concentration and the supply of NO_3_^−^ could be controlled (Figure 1). Preliminary attempts to incubate sediment in NO_3_^−^-amended anoxic seawater in batch mode failed due to substantial bubble formation in the sediment cores as a result of N_2_ production from denitrification. In November 2011, cylindrical glass chambers (inner diameter: 5.4 cm; height: 16 cm) were filled with sulfidic sediment up to ≈3 cm below the upper rim. The chambers were sealed with glass lids to be filled with water without leaving a gas phase, connected to the flow-through system and immersed into an aquarium containing anoxic water to assure that no O_2_ would diffuse through the sealing. Anoxic, NO_3_^−^-amended, artificial seawater (salinity: 30‰) based on MilliQ water (Millipore, Billerica, MA, USA) and Red Sea Salts (Red Sea Fish Pharm Ltd, Eilat, Israel) was pumped from a reservoir into the chambers at a constant rate of 190 μl min^−1^. Magnetic stir bars driven by an external rotating magnet maintained a homogeneous water column above the sediment in the chambers. The water in the reservoir was maintained at 30 °C and purged with N_2_ containing 0.04% CO_2_. Before entering the chambers, the water was cooled down to 13.6 °C to allow a constant exposure of the sediment to gas-undersaturated water, thereby preventing gas bubble formation. Throughout the 64 days of incubation, the NO_3_^−^ concentration in the inflowing and outflowing water was regularly monitored. The NO_3_^−^ concentration of the reservoir water remained constant within 266–273 μM.

Microprofiles of pH were measured in the sediment after 7, 18, 34, 53 and 64 days of incubation using pH microsensors. The depth distribution of NO_3_^−^+NO_2_^−^ (NO_x_^−^), O_2_ and H_2_S was measured at the end of the experiment using in-house made sensors for NO_x_^−^, O_2_ and H_2_S. The sediment cores were then sliced in 3 mm sections down to 18 mm depth. Each section was homogenously mixed and sediment samples (approx. vol. 1 ml) were collected in triplicates, transferred into polypropylene centrifuge tubes and frozen at −20 °C for later analysis of the intracellular+porewater NO_3_^−^ pool. This analysis was performed to address the presence of NO_3_^−^-storing organisms (for example, Foraminifera and *Beggiatoa*) in the sediment.

### Comparison of NO_3_^−^ and O_2_ effectiveness in sustaining distant sulfide oxidation

In March 2012, freshly collected sediment was pre-treated as described above and incubated in three treatments where the overlying artificial seawater was maintained aerated, anoxic (NO_3_^−^-free) or anoxic in the presence of 200 μM NO_3_^−^. The three parallel incubations lasted for 27, 24 and 28 days, respectively. In the anoxic incubations in presence of NO_3_^−^ (hereafter referred to as the NO_3_^−^ treatment), the sediment was packed into glass chambers and incubated in the flow-through system as described above. Additional sediment chambers (prepared as above, but not sealed at the top) were used for the oxic treatment. These chambers were immersed into an aquarium filled with artificial seawater, constantly flushed with air and maintained at 13.6 °C (Figure 1). Homogeneous water chemistry was maintained by a water pump placed in the aquarium and a magnetic stirrer suspended at about 2 cm above the sediment surface. In the anoxic (NO_3_^−^-free) control, the sediment was packed into glass liners (inner diameter: 1.8 cm; height: 10 cm) and placed into a sealed aquarium filled with artificial seawater at 13.6 °C. The water was kept stirred by means of an aquarium pump. Anoxic conditions (O_2_<0.1 μM) were maintained by constantly flushing the water with a gas mixture of N_2_ (99.96%) and CO_2_ (0.04%), and monitored throughout the entire incubation with the sensitive STOX O_2_ sensor (Revsbech *et al.*, 2009) inserted into the aquaria and connected to a strip chart recorder.

To evaluate whether cable bacteria can alternate between O_2_ and NO_3_^−^ as a terminal electron acceptor for driving distant sulfide oxidation, sediment cores previously incubated in the oxic treatment were exposed to NO_3_^−^-amended anoxic water. Hence, at the end of the oxic incubation the chambers were sealed at the top and connected to the flow-through system described above for the following 9 days. At the end of each incubation the vertical microdistribution of O_2_, NO_x_^−^, H_2_S and pH in the sediment was measured with microsensors.

### Microsensor measurements

Sediment microprofiles of H_2_S, O_2_ and pH were measured with microsensors (Revsbech and Jorgensen, 1986; Revsbech, 1989; Jeroschewski *et al.*, 1996), whereas microscale biosensors were prepared according to Revsbech and Glud (2009) for NO_x_^−^ (NO_3_^−^+NO_2_^−^). Total hydrogen sulfide (ΣH_2_S=[H_2_S]+[HS^−^]+[S^2−^]) concentrations were calculated at each depth from the measured H_2_S and pH values (Jeroschewski *et al.*, 1996). Microprofiles were measured by mounting single sensors on a computer-controlled microprofiler as described by Nielsen *et al.* (2009). Sensor tips were manually positioned at the sediment surface while observing them through a horizontal dissection microscope. Before measuring microprofiles in the NO_3_^−^ treatment, the water flow was stopped and the chambers removed from the anoxic aquarium. Microsensors were then inserted through openings (diameter: 1 cm) previously drilled into the glass lid (and maintained sealed during the incubation with rubber stoppers). To prevent O_2_ diffusion into the glass chamber, the openings were flushed with N_2_ during measurements. Consumption rates of the measured parameters were estimated by modeling the concentration microprofiles with the algorithm developed by Berg *et al.* (1998). Porosity (vol/vol) was determined from density and water content of 3-mm-thick sediment slides. The diffusion coefficients of O_2_, NO_3_^−^ and HS^−^ were calculated according to Boudreau (1997).

### Analysis of the intracellular+porewater NO_3_^−^ pool

The frozen sediment samples were thawed in boiling water to promote cell lysis (Risgaard-Petersen *et al.*, 2006) and then centrifuged at 2000 *g* for 10 min. The concentration of pooled intracellular and porewater-dissolved NO_3_^−^ was determined in the supernatant on a chemiluminescence detector (CLD 86, Eco Physics, Duernten, Switzerland) after being reduced to NO by the VCl_3_ method (Braman and Hendrix, 1989).

### Estimation of the cathodic O_2_ and NO_3_^−^ reduction rates and associated current densities

Minimum estimates of the cathodic O_2_ reduction and equivalent current density were calculated from the electron–proton–oxygen mass balance proposed by Nielsen *et al.* (2010).

Minimum estimates of the cathodic NO_3_^−^ reduction and equivalent current density were estimated from an electron–proton–nitrate mass balance. We considered two alternative cathodic NO_3_^−^ reductions that lead either to N_2_ (1) or to NH_4_^+^ (2) production:


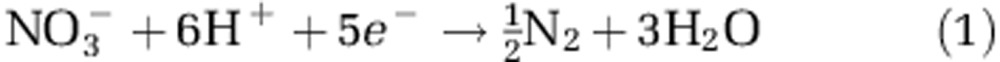



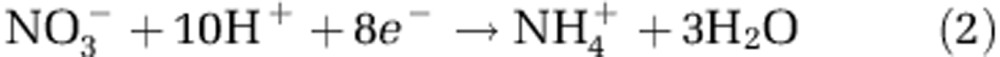


As main non-cathodic NO_3_^−^ reducing processes we considered organotrophic denitrification (3) and FeS oxidation with NO_3_^−^ (4).


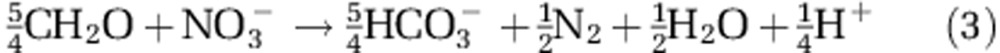



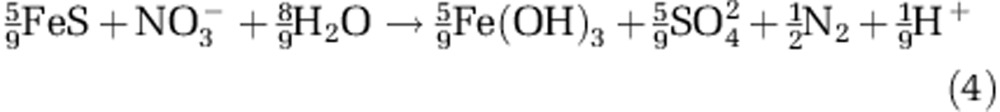


The net proton consumption in the NO_3_^−^ reduction zones (JH^+^) equals the proton consumption by cathodic NO_3_^−^ reduction minus the proton production due to the non-cathodic consumption of NO_3_^−^. This can be expressed as follows:


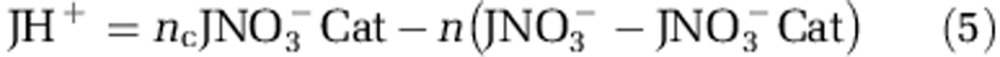


Here 
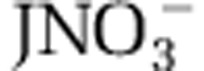
 is the total NO_3_^−^ consumption rate, 
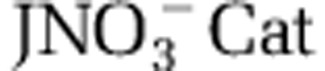
 is the rate of cathodic NO_3_^−^ reduction, *n*_c_ is the number of moles of protons consumed by one mole of NO_3_^−^ reduced cathodically and can be either 6 or 10 (Equations 1 and 2), *n* is the number of moles of protons produced by the non-cathodic reduction of one mole of NO_3_^−^ and can be either 1/4 or 1/9 (Equations 3 and 4). Rearranging Equation 5 gives the following expression for the rate of cathodic NO_3_^−^ reduction:


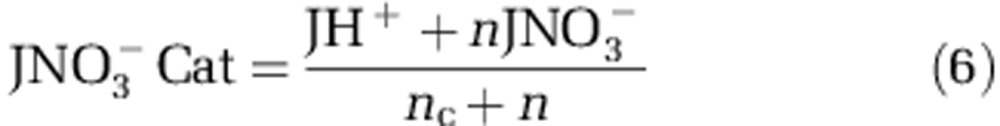


The cathodic NO_3_^−^ reduction rate was calculated for each of the four possible scenarios in which one of the two cathodic NO_3_^−^ reductions was alternatively assumed to compete with one of the two non-cathodic NO_3_^−^ reductions by substituting *n*_c_ and *n* according to the considered stoichiometry. JH^+^ in the NO_3_^−^ reduction zone was calculated on the basis of the pH microprofile and the dissolved inorganic carbon concentration in the sediment porewater according to Nielsen *et al.* (2010). Dissolved inorganic carbon was measured as CO_2_ after acidification on a gas chromatograph equipped with a thermal conductivity detector (ML GC 82, Mikrolab, Aarhus, Denmark). 
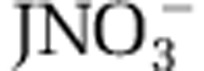
 was calculated as the net NO_3_^−^ flux across the water–sediment interface in the flow-through incubations by means of the following equation:


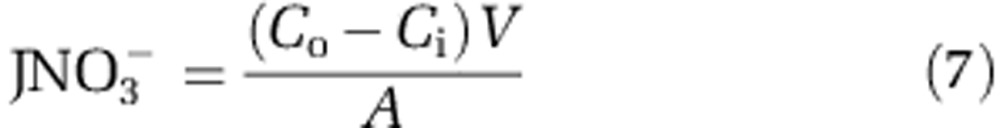


where *C*_o_ and *C*_i_ are the NO_3_^−^ concentrations in the water at the outlet and inlet of the chamber, respectively; *V* is the water flow rate and *A* is the surface area of the sediment core. The current density (J*e*^−^) needed to sustain the cathodic NO_3_^−^ reduction was calculated as follows:


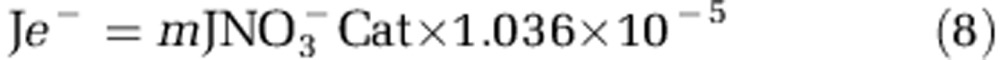


where *m* is the number of moles of electrons consumed by one mole of NO_3_^−^ reduced cathodically and can be either 5 or 8 (Equations 1 and 2), and 1.036 × 10^−5^ is the conversion factor from mol e^−^ s^−1^ to Ampere.

### Cable bacteria identification and density estimation

To investigate whether cable bacteria can grow by respiring NO_3_^−^ under anoxic conditions, freshly collected sediment was pre-treated as described above, packed into glass liners (inner diameter: 3.5 cm; height: 5.4 cm) and incubated in batch mode in anoxic, NO_3_^−^-amended seawater. Anoxic incubations without NO_3_^−^ served as controls. The O_2_ concentration in the overlying water (monitored with an O_2_ optode; Lumos, Graz University of Technology) was kept below 15 nM by continuous bubbling with N_2_/CO_2_ as described above, except for the short profiling periods, when O_2_ was up to 2.5 μM. Nitrate concentrations were kept at 80–350 μM (monitored as described above) by regularly adding NO_3_^−^ to the overlying water. Microprofiles of pH and H_2_S were recorded after 5, 10 and 12 days to confirm the establishment of electric coupling between NO_3_^−^ reduction and sulfide oxidation in the NO_3_^−^-amended cores. After 12 and 14 days, sediment cores were sectioned in 2 mm intervals down to 10 mm; sections were fixed in 50% ethanol (final concentration) and stored at −20 °C for fluorescence *in situ* hybridization (FISH), or snap frozen in liquid nitrogen and stored at −80 °C for DNA analysis. Control cores were sampled after 28 days.

DNA extraction (using PowerSoil DNA Isolation Kit (MO BIO Laboratories, Carlsbad, CA, USA)), PCR, cloning and sequence analysis were done as previously described (Pfeffer *et al.*, 2012), except that two different primer combinations were applied for PCR: (i) 8F (Loy *et al.*, 2002)/DSBB+1297R (Kjeldsen *et al.*, 2007); and (ii) ELF645F (5′-CTTGGCTTGAGTATCAGAGG-3′)/DSBB+1297R. The annealing temperature was 58 °C in both reactions. Sequences have been deposited at Genbank under accession number KJ021894 to KJ021926.

Identification and quantification of cable bacteria by FISH were done as previously described (Pfeffer *et al.*, 2012). Probes DSB706 (specific for *Desulfobulbaceae*) and ELF645 (specific for a *Desulfobulbaceae* lineage confirmed as cable bacteria) were applied to 6 × -diluted subsamples from 4–6 mm depth from NO_3_^−^-amended and control sediment of triplicate cores. Filament length density (that is, meters of filament per cubic centimeter of sediment) was determined for probe DSB706-positive filaments using the line-intercept method (Newman, 1966) as described for filamentous bacteria (Hogslund *et al.*, 2010). For comparison, filament length was directly measured in triplicates in digital images of at least 300 microscopic fields by microscope digital photography using imaging software (AxioVision, Carl Zeiss, Göttingen, Germany) (Schauer *et al.*, 2014). The fraction of ELF645-positive filaments was determined relative to all *Desulfobulbaceae* (probe DSB706-positive) filaments in triplicates by checking at least 1000 cells after double hybridizations with both probes, labeled in CY3 or FITC. Analyses were carried out on an Axiovert 200 M epifluorescence microscope (Carl Zeiss).

### Atomic force microscopy

The outer surface of cable bacteria was investigated by a combination of FISH and atomic force microscopy (AFM) imaging. Single filaments were picked with a sterile glass hook under microscopic guidance (Pfeffer *et al.*, 2012), cleaned in MilliQ water and transferred to gelatine-coated cover slides. Samples were dehydrated, and FISH was performed as described above with probe DSB706. Optical and AFM imaging were performed on a Zeiss Axiovert 200 M fluorescence microscope combined with a Nanowizard II AFM (JPK Instruments, Berlin, Germany). The coverslip was placed on the inverted microscope, and fluorescence images were obtained on the dry cells without any anti-bleaching agent using Zeiss filterset 43 to detect the CY3-labeled FISH probe. AFM images were then obtained from the same cells in ambient conditions using Olympus OMCL-AC160TS silicon cantilevers with a nominal spring constant of 26 N m^−1^ in intermittent contact mode at a target frequency of 332 Hz, target amplitude of 1.5 V, set-point value of 0.95 V and a scan rate of 1 Hz.

## Results

### Sediment geochemistry in the presence of NO_3_^−^ in overlying water

**Figure 2:**
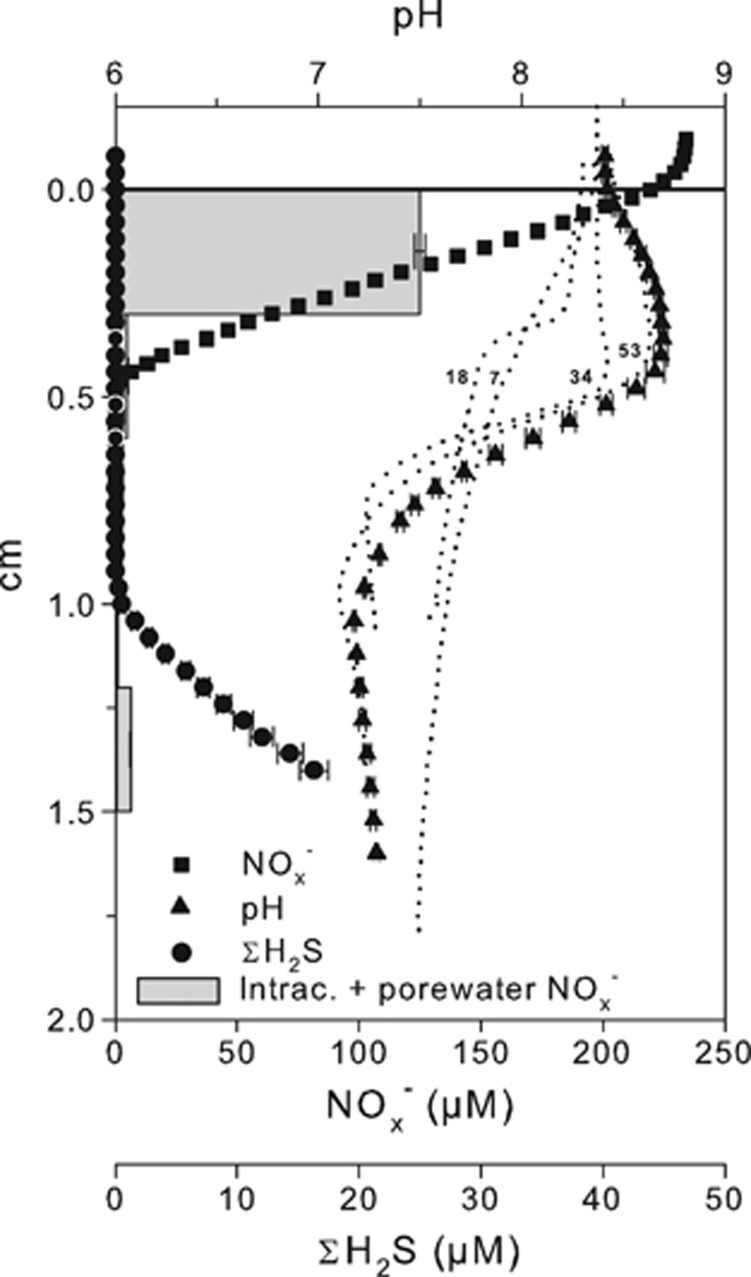
Microprofiles of pH, ΣH_2_S and NO_x_^−^ measured in sediment incubated for 64 days under anoxic overlying water in the presence of 235 μM NO_3_^−^ in November 2011. Data are shown as mean±s.e.m. (*n*=3). Dotted lines represent single pH profiles measured after 7, 18, 34 and 53 days of incubation. Gray bars represent pooled intracellular and free NO_3_^−^ extracted from frozen and thawed sediment samples. Data are shown as mean±s.e.m. (*n*=3).

Microdistributions of NO_x_^−^, ΣH_2_S and pH in sediment exposed to anoxic NO_3_^−^-amended seawater are shown in Figure 2. At the end of the incubation (Day 64), NO_x_^−^ penetrated 4–5 mm into the sediment, whereas ΣH_2_S was detectable from a depth of 9–10 mm, resulting in a 4–6-mm-thick zone devoid from both NO_x_^−^ and ΣH_2_S. Within the incubation time, the pH profile developed to a maximum at 4.2 mm and a minimum at 1 cm depth. The pH maximum and minimum indicated intense proton consumption and production at depths of NO_x_^−^ and ΣH_2_S consumption, respectively. Throughout the incubation, the pH minimum always coincided with the sulfide front (data not shown). The areal consumption rate of NO_x_^−^ was 118 and 104 μmol m^−2^ h^−1^ when calculated from microprofile modeling and from Equation 7, respectively. The ΣH_2_S oxidation rate derived from microprofile modeling was 11.9 μmol m^−2^ h^−1^.

Pooled intracellular and porewater NO_3_^−^ profile showed concentrations comparable to those measured with the NO_x_^−^ microsensor in the depth interval 0–3 mm. Below 3 mm depth, the concentration decreased to insignificant values, indicating the absence of NO_3_^−^-storing organisms.

### Sediment geochemistry in the presence or absence of O_2_ and NO_3_^−^ in overlying water

**Figure 3:**
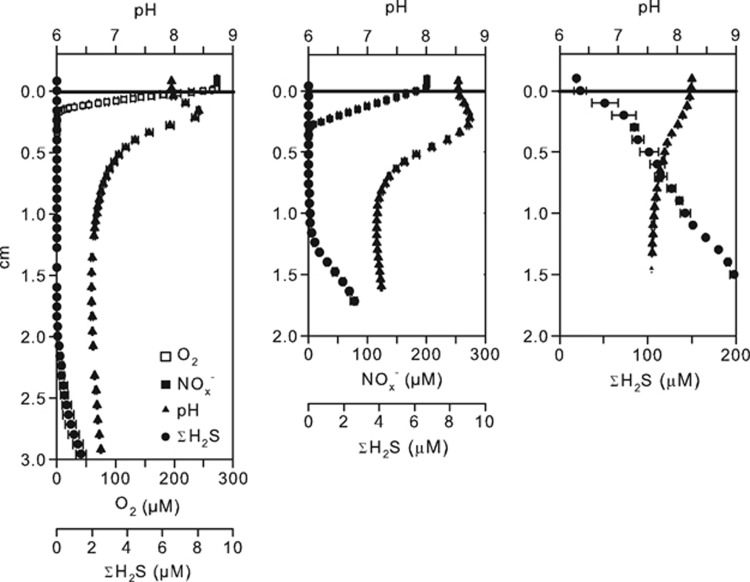
Sediment microprofiles of pH, ΣH_2_S, O_2_ and NO_x_^−^ measured in sediment cores incubated in March 2012 under oxic (left panel), anoxic 200 μM NO_3_^−^-amended (center panel) and anoxic NO_3_^−^-free (left panel) overlying water.

**Figure 4:**
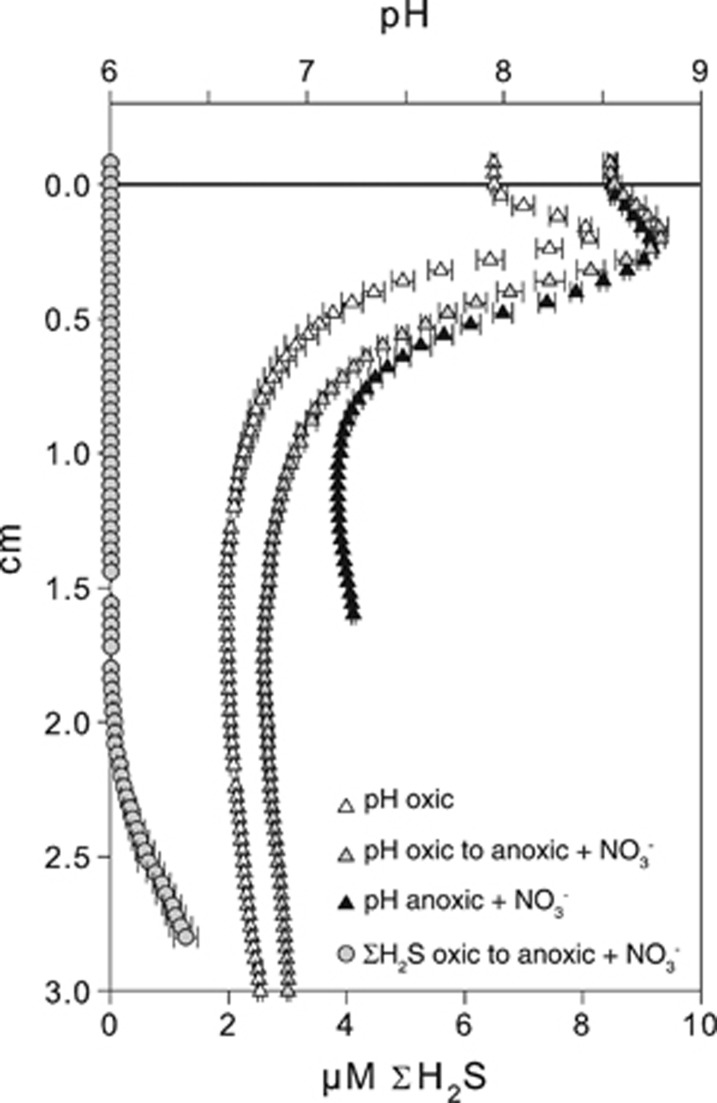
(Gray) Microprofiles of pH (triangle) and ΣH_2_S (circles) measured in cores incubated under oxic overlying water for 27 days and successively exposed to anoxic overlying water containing 200 μM NO_3_^−^ for 9 days (oxic to anoxic+NO_3_^−^). For comparison, pH microprofiles measured in the same core before the change in overlying water conditions (open triangles) (oxic) and pH microprofiles measured in cores only exposed to the NO_3_^−^ treatment (black triangles) (anoxic+NO_3_^−^) are shown (these profiles correspond to the profiles shown in Figure 3). Data are shown as mean±s.e.m. (*n*=3).

Microdistribution of O_2_, ΣH_2_S, NO_x_^−^ and pH in three parallel incubations where the sediment was exposed to oxic, anoxic NO_3_^−^-amended and anoxic NO_3_^−^-free seawater are shown in Figure 3. Sediment incubated with anoxic NO_3_^−^-free overlying water remained fully sulfidic and the pH decreased along with depth after 24 days of incubation. In the NO_3_^−^ treatment, ΣH_2_S was progressively consumed from the surface sediment, and at the end of the incubation (Day 28) the sulfidic front was detected at a depth of 11 mm. Nitrate penetrated 3.8 mm into the sediment and a distinct pH peak indicated proton consumption in the NO_3_^−^ reduction zone. The estimated current density in the NO_3_^−^ treatment varied between 6.6 and 8.8 mA m^−2^ depending on whether N_2_ or NH_4_^+^ was considered as the cathodic end product and whether organic carbon or FeS was the major electron donor for non-cathodic NO_3_^−^ reduction. The estimated cathodic NO_3_^−^ reduction represented only 12 and 19% of the total NO_3_^−^ consumption. In the oxic treatment, ΣH_2_S was detected at 21 mm depth after 27 days of incubation. Oxygen penetrated 1.8 mm into the sediment resulting in a ≈19-mm-thick suboxic zone. The pH microprofile showed a peak at a depth of 1.8 mm indicating net proton consumption. The cathodic O_2_ reduction accounted for 34% of the total O_2_ consumption supporting a current density of 28 mA m^−2^. Both the pH peak and the sulfide-free zone remained 9 days after the overlying oxic water was replaced with NO_3_^−^-amended anoxic water (Figure 4).

### Filaments identification and density

**Figure 5:**
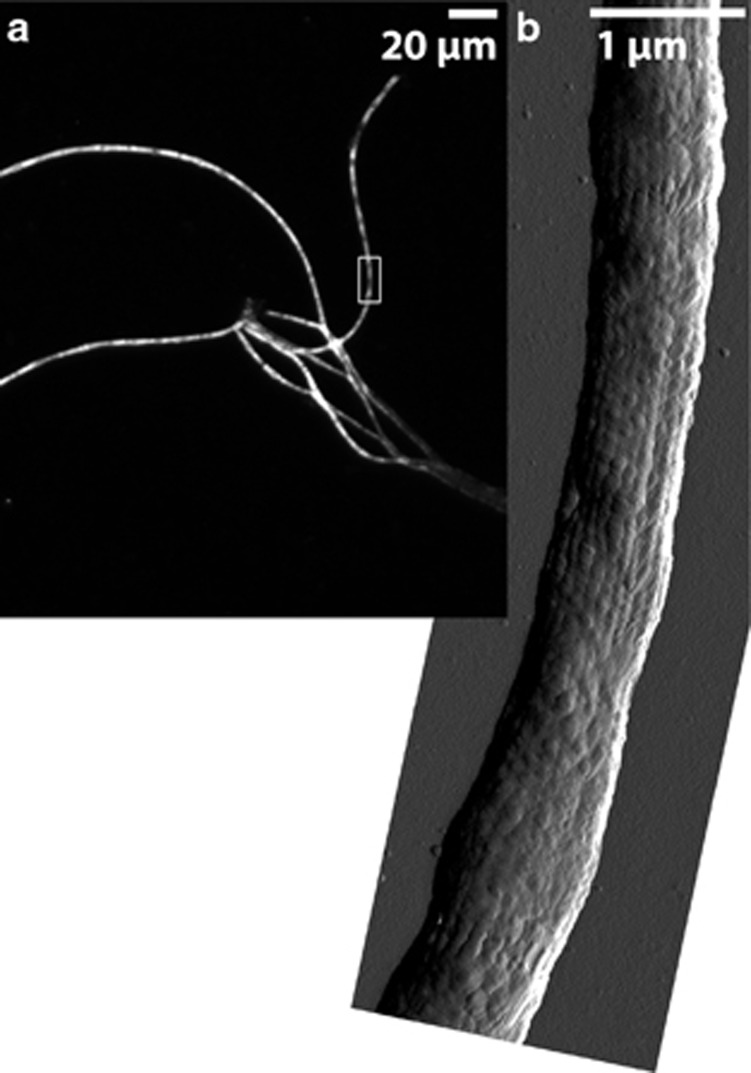
(**a**) Fluorescence *in situ* hybridization micrograph of filamentous *Desulfobulbaceae* identified by probe DSB706. (**b**) The characteristic ‘cable-like' structure of these bacteria is confirmed in the AFM amplitude image.

In the batch incubation with NO_3_^−^, the ΣH_2_S front retracted from 2 mm (Day 5) to 4.7 mm (Day 10) to 7.6 mm (Day 12), and a pH peak appeared at 1.6 mm depth at day 12. The controls remained fully sulfidic and no pH peak developed (data not shown). Filamentous *Desulfobulbaceae* similar to the previously described cable bacteria (Pfeffer *et al.*, 2012) were detected by FISH with probe DSB706 (Figure 5a). Filament length density at 4–6 mm depth was 30±7 m cm^−3^ (mean of triplicate cores±s.d.) by the line-intercept method and 85±22 m cm^−3^ by direct measurement in the NO_3_^−^-amended sediment. Filament diameters ranged from 0.4 to 1.0 μm, with an average of 0.6 μm. Only a few and rather short filaments were detected in the controls, with a length density <3 m cm^−3^. A filament subpopulation hybridized with the more specific probe ELF645 (Figure 5a); these cable bacteria accounted for 21.2% (±12.6%) of all DSB706-positive filamentous *Desulfobulbaceae*. Likewise, only 5 out of 33 *Desulfobulbaceae*-like 16S rRNA gene sequences clustered with the previously described ‘cable bacteria lineage' (Pfeffer *et al.*, 2012) (Supplementary Figure S1).

Longitudinal structures along the filaments similar to those observed by Pfeffer *et al.* (2012) were identified for filamentous bacteria from NO_3_^−^-treated sediment, using combined optical and AFM imaging (Figure 5b). These filaments were first hybridized with probe DSB706 to confirm their affiliation with *Desulfobulbaceae* (Figure 5a).

## Discussion

### Electric coupling between NO_3_^−^ reduction and ΣH_2_S oxidation

The results of this study show that NO_3_^−^ reduction can sustain the distant oxidation of ΣH_2_S in marine sediment as previously described for O_2_ (Nielsen *et al.*, 2010). Exposing the sediment to NO_3_^−^-amended anoxic overlying water resulted in the development of geochemical traits typical of electric coupling between distant half-cell reactions such as: (1) development of a 4–7-mm-wide zone devoid of both NO_3_^−^ and ΣH_2_S, consistent with the separation of ΣH_2_S oxidation from the associated NO_3_^−^ reduction; (2) consumption of protons in the NO_3_^−^ reduction zone and proton production at the ΣH_2_S oxidation depth consistent with the presence of cathodic NO_3_^−^ reduction and anodic ΣH_2_S oxidation, respectively. Furthermore, sediment that showed the geochemical traits typical of electric coupling between distant half-cell reactions in the oxic treatment maintained those characteristics after O_2_ was replaced with NO_3_^−^ (Figure 4). Previous investigations of similar sediment showed a fast rising of the ΣH_2_S front (up to 5 mm d^−1^) as a consequence of the interruption of the electric current either by physically disturbing the sediment (Pfeffer *et al.*, 2012) or by removing O_2_ from the overlying water (Nielsen *et al.*, 2010). The stability of the ΣH_2_S front combined with the presence of marked proton consumption in the upper sediment indicates that NO_3_^−^ can efficiently drive the distant oxidation of ΣH_2_S when O_2_ is no longer available.

Large sulfur bacteria of genus *Beggiatoa* are able to transport NO_3_^−^ from the sediment surface and use it to oxidize the ΣH_2_S, with the resultant formation of a suboxic zone in marine sediments (for example, Sayama *et al.* (2005)). We can exclude that *Beggiatoa* played a significant role in the separation of NO_3_^−^ and ΣH_2_S in our incubations. Because of their ability to accumulate NO_3_^−^ intracellularly to millimolar concentrations (Jørgensen and Nelson, 2004), intracellular NO_3_^−^ measured in sediment inhabited by these organisms largely exceed the concentration of NO_3_^−^ dissolved in the porewater (Sayama, 2001; Preisler *et al.*, 2007). In our incubation, the pooled intracellular and porewater NO_x_^−^ profile agreed with the porewater NO_x_^−^ microprofile measured by the biomicrosensor, indicating that intracellular NO_3_^−^ storage was insignificant (the low NO_3_^−^ concentration values detected below 6 mm depth were likely due to analytical errors or contaminations). Moreover, the activity of *Beggiatoa* would result in a characteristic pH microprofile with a minimum in the upper sediment and a maximum at the ΣH_2_S front (Sayama *et al.*, 2005) and our pH microprofiles showed opposite trends.

The reduction of solid Fe and Mn oxides coupled to oxidation of ΣH_2_S can also result in the development of suboxic zones in sediments. Reactive fractions of Fe and Mn oxides are not expected to be present in our incubations, as the batch sediment was highly sulfidic and bioturbating infauna possibly mediating their formation was absent. In addition, in the anoxic NO_3_^−^-free treatment the sediment remained fully sulfidic and the pH did not show defined peaks, confirming that the background concentrations of solid electron acceptors were neither sufficient to generate a ΣH_2_S-free zone nor to sustain an intense proton-consuming process.

### Filamentous Desulfobulbaceae

The presence of filamentous *Desulfobulbaceae* resembling the cable bacteria described by Pfeffer *et al.* (2012) in NO_3_^−^-amended anoxic sediment and their absence in the NO_3_^−^-free anoxic incubations indicates that these organisms can grow using NO_3_^−^ as an electron acceptor in the absence of O_2_. The presence of geochemical traits indicative of electric coupling between NO_3_^−^ reduction and ΣH_2_S oxidation in NO_3_^−^-amended cores with cable bacteria further suggests that these organisms can perform cathodic NO_3_^−^ reduction and mediate an electric coupling between distant NO_3_^−^ reduction and ΣH_2_S oxidation in the absence of O_2_. However, NO_3_^−^ seemed to be a much less effective electron acceptor as compared with O_2_. The current density generated with NO_3_^−^ acting as electron acceptor was less than 9 mA m^−2^, which was less than one-third of the current density estimated in parallel oxic incubations (28 mA m^−2^). Interestingly, only a fraction of all filamentous *Desulfobulbaceae* (detected by probe DSB706) hybridized with probe ELF645 specifically designed for cable bacteria (Pfeffer *et al.*, 2012). This result indicates that probe ELF645 does not target all cable bacteria and suggests a broader diversity of cable bacteria within the *Desulfobulbaceae*. This conclusion is supported by the 16S rRNA gene sequence analysis that showed a dominance of diverse *Desulfobulbaceae* sequences outside the previously described cable bacteria cluster (Supplementary Figure S1). The true extent of the cable phenotype within *Desulfobulbaceae* remains yet to be defined.

In the present study we have demonstrated that cathodic NO_3_^−^ reduction can sustain the distant oxidation of ΣH_2_S in marine sediment as previously described for O_2_, and that cable bacteria are involved. The ability of microorganisms to perform cathodic NO_3_^−^ reduction has been previously invoked to explain electric current generation in microbial fuel cells (for example, Gregory *et al.* (2004)) and syntrophic relations in pure cultures in the presence of magnetite (Kato *et al.*, 2012). For these organisms, cathodic NO_3_^−^ reduction implies electron transfer from a cell to an external electrode or a conductive mineral. For cable bacteria, cathodic NO_3_^−^ reduction implies the transfer of electrons from distant donors through internal biological structures over millimeter to centimeter distances. The importance of this hitherto unknown mechanism for elemental cycling remains to be addressed.
