# Supplementary material for: Electric coupling between distant nitrate reduction and sulfide oxidation in marine sediment
Source: ISME J. 2014 Feb 27;8(8):1682–90. doi: 10.1038/ismej.2014.19 (PMC4817607; doi:10.1038/ismej.2014.19)
Supplement: Supplementary Information [file ismej201419x2.pdf]

Figure S1. Phylogenetic affiliation of 16S rRNA gene sequences retrieved during this study from anoxic, nitrate-amended sediment with electric coupling of nitrate reduction and sulfide oxidation (blue and red sequences). Numbers in brackets indicate the number of clones.

Sequences were aligned using the SINA online tool (Pruesse et al., 2012) and added to the Silva Release 115 SSU Ref database (Quast et al., 2013) using ARB. The tree was calculated by the maximum likelihood method implemented in ARB (Ludwig et al., 2004); filled and open circles indicate bootstrap support (by rapid bootstrap analysis implemented in ARB; 1000 iterations) ≥ 80% and ≥ 50%, respectively.

Sequences carrying the target site for probes DSB706 or ELF645 are indicated by the green or yellow shading, respectively. Sequences displayed in red were obtained with primer pair ELF645F (5' - CTT GGC TTG AGT ATC AGA GG – 3') and DSBB+1297R (Kjeldsen et al., 2007); it is thus not possible to confirm whether they have a perfect match to probe ELF645. Sequences displayed in blue were obtained with primer pair 8F (Loy et al., 2002) and DSBB+1297R.

References

Kjeldsen, K.U., Loy, A., Jakobsen, T.F., Thomsen, T.R., Wagner, M., and Ingvorsen, K. (2007) Diversity of sulfate-reducing bacteria from an extreme hypersaline sediment, Great Salt Lake (Utah). *FEMS Microbiology Ecology* **60**: 287-298.

Loy, A., Lehner, A., Lee, N., Adamczyk, J., Meier, H., Ernst, J. et al. (2002) Oligonucleotide microarray for 16S rRNA gene-based detection of all recognized lineages of sulfate-reducing prokaryotes in the environment. *Applied and Environmental Microbiology* **68**: 5064-5081.

Ludwig, W., Strunk, O., Westram, R., Richter, L., Meier, H., Yadhukumar et al. (2004) ARB: a software environment for sequence data. *Nucleic Acids Research* **32**: 1363-1371.

Pruesse, E., Peplies, J., and Glöckner, F.O. (2012) SINA: Accurate high-throughput multiple sequence alignment of ribosomal RNA genes. *Bioinformatics* **28**: 1823-1829.

Quast, C., Pruesse, E., Yilmaz, P., Gerken, J., Schweer, T., Yarza, P. et al. (2013) The SILVA ribosomal RNA gene database project: improved data processing and web-based tools. *Nucleic Acids Research* **41**: D590-D596.
